# Diabetic Peripheral Neuropathy With Low Appendicular Muscle Mass Is Associated With Osteoporosis, Falls, and Fractures

**DOI:** 10.1111/1753-0407.70276

**Published:** 2026-10-28

**Authors:** Biao Zheng, Yongze Zhang, Dan Liang, Ximei Shen, Lingning Huang, Fengying Zhao, Sunjie Yan

**Affiliations:** ^1^ Department of Endocrinology The First Affiliated Hospital, Fujian Medical University Fuzhou China; ^2^ Department of Endocrinology, National Regional Medical Center Binhai Campus of the First Affiliated Hospital, Fujian Medical University Fuzhou China; ^3^ Clinical Research Center for Metabolic Diseases of Fujian Province The First Affiliated Hospital, Fujian Medical University Fuzhou China; ^4^ Fujian Key Laboratory of Glycolipid and Bone Mineral Metabolism The First Affiliated Hospital, Fujian Medical University Fuzhou China; ^5^ Diabetes Research Institute of Fujian Province The First Affiliated Hospital, Fujian Medical University Fuzhou China; ^6^ Metabolic Diseases Research Institute, the First Affiliated Hospital, Fujian Medical University Fuzhou China

**Keywords:** diabetic peripheral neuropathy, falls, fractures, low appendicular muscle mass, osteoporosis

## Abstract

**Introduction:**

Diabetic peripheral neuropathy (DPN) and low appendicular muscle mass (LAMM) may jointly contribute to adverse skeletal outcomes, but their combined effects remain unclear. We examined their associations with osteoporosis, falls, and fractures in patients with Type 2 diabetes.

**Methods:**

We explored associations of diabetes and grip strength with skeletal outcomes using SHARE v8.0.0 (327 248 participants). The primary analysis used a cohort of patients with Type 2 diabetes from the First Affiliated Hospital of Fujian Medical University. LAMM was defined using dual‐energy X‐ray absorptiometry‐derived appendicular skeletal muscle mass index. Associations of clinically diagnosed DPN, LAMM, and their coexistence with osteoporosis, falls, and fractures were evaluated using logistic regression and Cox survival models. Exploratory serum antibody omics was performed.

**Results:**

In SHARE, grip strength was inversely associated with all three outcomes. In the local cohort, coexisting DPN and LAMM were associated with higher odds of osteoporosis (OR 2.151, 95% CI 1.645–2.805), falls (OR 4.667, 95% CI 3.406–6.376), and fractures (OR 2.416, 95% CI 1.367–3.515; all *p* < 0.001). Cox analyses showed higher hazards of osteoporosis (HR 3.410, 95% CI 1.579–7.362; *p* = 0.002) and falls (HR 4.385, 95% CI 1.838–10.459; *p* < 0.001), but not fractures (HR 0.708, 95% CI 0.187–2.675; *p* = 0.611). Antibody omics suggested a reactivity profile involving endoplasmic reticulum protein processing and neurodegeneration‐related pathways.

**Conclusion:**

Coexisting DPN and LAMM were associated with higher odds of all three skeletal outcomes and higher longitudinal hazards of osteoporosis and falls, but not fractures. The exploratory omics findings require functional validation.

## Highlights

- Clinically diagnosed DPN combined with LAMM was associated with higher risks of osteoporosis, falls, and fractures.
- The coexistence of DPN and LAMM showed stronger associations with adverse skeletal outcomes than either condition alone.
- The omics findings provided preliminary pathway‐level clues but require further functional validation.

## Graphical abstract

Diabetics have higher risks of osteoporosis, falls, and fractures; higher grip strength predicts lower risks. In both cross‐sectional and longitudinal data, diabetic patients with both peripheral neuropathy and low appendicular muscle mass have the highest risks of osteoporosis and falls.

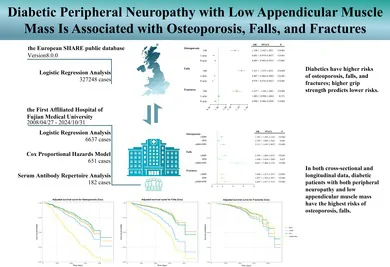

**Abbreviations**
ACEI/ARB: Angiotensin‐Converting Enzyme Inhibitors or Angiotensin II Receptor Blockers
ALB: albumin
BF: body fat ratio
BH: Benjamini–Hochberg
BMD: bone mineral density
BMI: body mass index
CCB: calcium channel blockers
CR: creatinine
CTX: type i collagen cross‐linked C‐telopeptide
DF: diabetic foot
DKD: diabetic kidney disease
DM: diabetes mellitus
DPP4i: dipeptidyl peptidase‐4 inhibitors
DR: diabetic retinopathy
eGFR: estimated glomerular filtration rate
ER: endoplasmic reticulum
FC: fold change
FDR: false discovery rate
GLP1ra: glucagon‐like peptide‐1 receptor agonists
GLU: glucose
HbA1c: glycated hemoglobin A1c
HT: hypertension
KEGG: Kyoto Encyclopedia of Genes and Genomes
LAMM: low appendicular muscle mass
logCPM: log_2_ counts per million
OP: Osteoporosis
PhIP‐Seq: phage immunoprecipitation sequencing
SGLT2i: sodium‐glucose linked transporter 2 inhibitors
TC: total cholesterol
TG: triglycerides
Total PINP: type I procollagen N‐terminal propeptide
UACR: urinary albumin‐to‐creatinine ratio
VD: 25‐hydroxyvitamin D
WHtR: waist‐to‐height ratio

## 1 Introduction

Over the past 30 years, the number of people with diabetes has increased significantly, and the prevalence rate is still rising [1]. The 2024 grassroots diagnosis and treatment guidelines mentioned that the prevalence of diabetes among adults over the age of 18 in China has reached 11.2% [2]. Subsequently, the complications of diabetes also bring people great trouble [3]. Diabetic Peripheral Neuropathy (DPN) is one of the common complications of diabetes and plays an important role in persons' falls and fractures [2], with extremely high mortality and disability rates [4].

At present, the problem of population aging is intensifying, and sarcopenia (SP), as an age‐related disease [5, 6], is also attracting more and more attention. Osteoporosis (OP), another disease that mostly affects middle‐aged and elderly people, is not only negatively affected by diabetes, but is also closely related to neuromuscular diseases [7]. Sarcopenia is positively correlated with falls and fractures [5, 8, 9], and having osteoporotic sarcopenia is a risk factor for falls [10].

Previous studies have indicated that DPN is closely associated with sarcopenia [11], and impaired peripheral nerve function may be correlated with lower bone mineral density values [12]. However, sarcopenia is a multidimensional muscle disorder that generally requires assessment of muscle mass together with muscle strength and/or physical performance. In routine clinical practice, patients with diabetes frequently present with reduced appendicular muscle mass, but handgrip strength and gait speed are not always available in hospital‐based databases. Therefore, rather than defining sarcopenia solely on the basis of ASMI, the present study focused on ASMI‐defined low appendicular muscle mass (LAMM) as a measurable muscle‐mass phenotype. We aimed to investigate whether DPN combined with LAMM was associated with osteoporosis, falls, and fractures in patients with Type 2 diabetes.

## 2 Materials and Methods

### 2.1 Study Population

We first conducted a preliminary experiment and used the European SHARE public database [13, 14] (Version8.0.0, 327248 cases) to initially explore the potential relationship between diabetes, grip strength and three events: osteoporosis, falls and fractures. Subsequently, a cross‐sectional study was conducted at The First Affiliated Hospital of Fujian Medical University from April 27, 2008, to October 31, 2024. Initially, 9661 cases were enrolled. After excluding cases with incomplete data, a total of 6637 patients with complete database was included for final analysis. This study aimed to investigate the prevalence of osteoporosis, falls, and fractures among patients presenting with comorbid DPN and LAMM. Based on hospitalization records indicating whether outcome events occurred after the initial follow‐up, a total of 651 patients were identified and constituted the follow‐up cohort. This cohort study design was used to further validate the findings from the cross‐sectional study. Finally, serum samples were collected from participating patients on a voluntary basis for serum proteomics analysis. This study has been approved by the Ethics Committee of The First Affiliated Hospital of Fujian Medical University (MRCTA, ECFAH of FMU, 2017‐131), and written informed consent was obtained from all participants.

For the public database, indicators irrelevant to this study were removed, and missing values were imputed using the random forest multiple imputation method. For the local database, set inclusion criteria: (1) Patient meets diagnostic criteria for Type 2 diabetes [15]; (2) Patients have complete bone mineral density (BMD), body composition, laboratory and other examination data. Set exclusion criteria: (1) Patients with Type 1 diabetes, gestational diabetes, and other special types of diabetes; (2) The patient was in a state of acute diabetic complications, such as diabetic ketoacidosis (DKA), hyperosmolar hyperglycemic state (HHS), or hypoglycemia; (3) Systemic diseases such as severe infections, tumors, and severe malnutrition; (4) Secondary osteoporosis, such as hyperparathyroidism, chronic kidney disease, and so forth [16]; (5) Athletes or pregnant women.

### 2.2 Research Methods

#### 2.2.1 Public Database

We applied and downloaded the European SHARE database through official channels, deleting indicators that are not relevant to this study. The SHARE database was used only as an exploratory population‐level analysis to examine whether diabetes and reduced grip strength were associated with osteoporosis, falls, and fractures. Because SHARE did not provide sufficient information to define diabetic peripheral neuropathy and DXA‐based sarcopenia according to the criteria used in the local cohort, it was not used as an external validation cohort for the main hypothesis regarding comorbid DPN and sarcopenia.

#### 2.2.2 Local Database

The Diabetic Database of the Department of Endocrinology, The First Affiliated Hospital of Fujian Medical University, hereinafter referred to as the Local Database.

#### 2.2.3 History Taking and Physical Examination

We use the patient's hospitalization number and ID number to search cases, record basic information such as age and gender, and manually proofread it to establish a database. For patients with two hospitalizations, their first and last hospitalizations were annotated to form a follow‐up database. All patients underwent a comprehensive physical examination, including but not limited to the collection of height, weight, waist circumference, hip circumference, and other information. Measurement of height and weight: getting up in the morning on an empty stomach, after emptying urine, the patient wore light clothes and stood barefoot on the measuring instrument (type: RGZ‐120‐RT), with both heels together, heel, sacrum, and shoulders close to the post of the altimeter. Waist circumference and hip circumference measurement: the patient wears light clothing, and the upper limbs are naturally abducted at an angle of about 45°. Take 2 cm above the navel plane and use a soft ruler to surround the skin as closely as possible without pulling the soft ruler; then ask the patient to stand upright with legs together, the upper limbs drooping naturally, take the most prominent part of the buttocks, and circle around the horizontal plane through the pubic symphysis. Systolic blood pressure (SBP) and diastolic blood pressure (DBP) were measured after resting for at least 15 min. BMI equals weight (kg) divided by height squared (m^2^). The waist‐to‐height ratio (WHtR) is calculated by dividing the waist circumference (cm) by the height (cm).

#### 2.2.4 BMD And Body Composition Examination

Bone mineral density (BMD) was measured using dual‐energy X‐ray absorptiometry (GE LUNAR company, prodigy). The DXA system was calibrated annually using a standardized model (1.000 g/cm^2^) to maintain accuracy, and the following parameters were recorded: appendicular skeletal muscle mass (ASM), total body fat mass (TBFM), lumbar spine bone mineral density (L1‐4 BMD), left femoral neck BMD (FNBMD), and left hip BMD. The appendicular skeletal muscle mass index (ASMI) was calculated as ASM divided by height in meters squared (m^2^) [6]. Body fat ratio (BF) is equal to TBFM (kg) as a percentage (%) of total body weight (kg) [17].

#### 2.2.5 Clinical Biochemical Examination

All patients began fasting and abstaining from water at night, and venous blood samples were collected the next morning. In the blood samples, albumin (ALB), total cholesterol (TC), triglycerides (TG), creatinine (CR), estimated glomerular filtration rate (eGFR), natrium (Na), kalium (K), calcium (Ca), 25‐hydroxyvitamin D (VD), Type I procollagen N‐terminal propeptide (total PINP), and Type I collagen cross‐linked C‐telopeptide (CTX) were all measured using the German Siemens ADVIA 2400 fully automated biochemical analyzer. Among these, eGFR was automatically calculated by the instrument based on the patient's age, gender, and CR value. Glycated hemoglobin A1c (HbA1c) was measured using the Variant II hemoglobin testing system. In the morning urine samples, the urinary albumin‐to‐creatinine ratio (UACR) was measured.

### 2.3 Diagnosis and Definition

#### 2.3.1 Clinically Diagnosed Diabetic Peripheral Neuropathy (DPN)

DPN is a group of clinical syndromes with diverse manifestations, which can cause partial nerve dysfunction and/or physical symptoms, and abnormalities may also be detected during clinical physical examinations [2]. In the present study, DPN refers to clinically diagnosed DPN, which was defined according to “Guideline for the prevention and treatment of diabetes mellitus in China (2024 edition)” [1], the diagnostic criteria are as follows: ① A clear history of diabetes. ② Onset of the condition at the time of or after the diabetes diagnosis. ③ Presence of clinical symptoms of neuropathy, such as pain, numbness, or paresthesia, with any one abnormality found in the five tests (ankle reflex, vibration sensation, pressure sensation, temperature sensation, pinprick sensation); if no clinical symptoms are present, diagnosis can also be made if any two of the five tests are abnormal. ④ Exclusion of other causes of neuropathy, including neurotoxic drugs, vitamin B12 deficiency, cervical/lumbar spondylosis, cerebral infarction, chronic inflammatory demyelinating polyneuropathy, hereditary neuropathies, and nerve damage caused by metabolic toxins resulting from vasculitis, infection, or renal insufficiency. If the diagnosis cannot be confirmed based on the above assessments, the patient will be referred to a neurologist for neurophysiological testing and diagnosis. Because nerve conduction studies were not routinely available for all participants, the DPN definition used in this study should be interpreted as clinically diagnosed DPN rather than electrophysiologically confirmed DPN.

#### 2.3.2 Low Appendicular Muscle Mass (LAMM)

The European Working Group on Sarcopenia in Older People (EWGSOP) first proposed a definition in 2010, identifying sarcopenia as an aging‐related syndrome characterized by decreased muscle mass, reduced muscle strength, and/or diminished physical performance [5]. The diagnostic criteria of the International Working Group on Sarcopenia (IWGS) are: (i) gait speed less than 1.0 m/s, and (ii) ASMI ≤ 7.23 kg/m^2^ for men and ASMI ≤ 5.67 kg/m^2^ for women [6]. Other working groups have incorporated grip strength to comprehensively assess the condition [18]. However, due to the difficulty of conducting these tests and low patient compliance, our hospital currently does not record these two items. Therefore, in this study, LAMM was defined as ASMI ≤ 7.23 kg/m^2^ in men and ≤ 5.67 kg/m^2^ in women, according to the IWGS‐referenced cut‐off values for LAMM. Throughout this study, the term “LAMM” refers specifically to reduced ASMI and should not be interpreted as a complete diagnosis of sarcopenia.

#### 2.3.3 Osteoporosis

Osteoporosis is a systemic bone disease characterized by low bone mass, damage to bone tissue microstructure, resulting in increased bone fragility and susceptibility to fractures [7]. According to the standards formulated by the World Health Organization (WHO) and domestic and foreign guidelines, we select the bone mineral density of the axial bone (PA spine, hip, femoral neck) as the basis. If at least one site is lower than the average value of the same age group by 2.5SD or above (T‐score ≤ 2.5), the diagnosis is established [7, 19].

#### 2.3.4 Risk of Falls and Fractures

The risk of falls is quantified using the Morse Fall Scale, with a score greater than 45 indicating a high risk of falls [20, 21]. Fracture risk assessment consists of three components: bone density test results, the FRAX tool, and major osteoporotic fractures. Based on this assessment, individuals are categorized into low‐risk, moderate‐risk, and high‐risk groups. Additionally, a very high‐risk group is included in this study and classified under the high‐risk category [22]. In all statistical analyses, including the longitudinal analyses, falls and fractures were defined as documented clinical events rather than the risk categories described above.

### 2.4 Serum Antibody Omics

All serum samples were of human origin, collected with informed consent and properly documented. After centrifugation, the serum samples were aliquoted into EP tubes and stored at −80°C until analysis. In this study, serum antibody omics data were obtained from an established phage immunoprecipitation sequencing (PhIP‐Seq)‐based antibody profiling platform developed by our research group. The local database cohort was matched with the existing biobank registry, and 182 patients with both complete clinical data and available serum samples were identified for serum antibody omics analysis. According to the presence or absence of DPN and LAMM, the samples were divided into four groups: None, LAMM, DPN, and LAMM + DPN, with a distribution of 108, 60, 9, and 5 cases, respectively.

The antibody profiling library contained 470 850 peptides. Briefly, diluted serum samples were incubated with the phage‐displayed peptide library to allow antibody–peptide binding. Immune complexes were subsequently captured using Protein G Dynabeads, followed by repeated washing, elution, PCR amplification, and high‐throughput sequencing on the Illumina HiSeq platform. Positive and negative controls were included during the immunoprecipitation procedure to ensure assay reliability.

After sequencing, paired‐end reads were subjected to quality control and bioinformatic processing. Only insert sequences with perfect concordance between paired‐end reads and no mismatches were retained for downstream analysis. Each insert contained sample‐specific barcode sequences and a 36‐bp variable DNA region, which was translated in silico into a 12‐amino acid peptide sequence. Peptide sequences were then assigned to corresponding serum samples according to the dual‐barcode system, and peptide‐level read counts were quantified for each sample.

For differential antibody reactivity analysis, peptide counts were normalized before intergroup comparison. Differentially reactive peptides among the four clinical groups were screened using the Wilcoxon rank‐sum test. For each peptide, fold change, raw *p* value, and false discovery rate (FDR)‐adjusted *q* value were calculated. Considering the large‐scale screening of 470 850 peptides, *p* values were adjusted using the Benjamini–Hochberg procedure to control the FDR. Differential peptides were defined as those with an absolute fold change > 2 and an FDR‐adjusted *q* value < 0.05. Peptides with nominal *p* < 0.05 but *q* ≥ 0.05 were considered exploratory and were not used as the primary basis for statistical significance.

Differential peptides were mapped to the human proteome using BLASTP against UniProtKB. For proteins matched by multiple peptides, the peptide with the largest absolute log_2_ fold change was selected to represent differential antibody reactivity. Gene Ontology (GO) and Kyoto Encyclopedia of Genes and Genomes (KEGG) enrichment analyses were performed using the clusterProfiler package to explore the potential biological significance of the identified proteins. Enrichment results were also adjusted for multiple comparisons using the Benjamini–Hochberg procedure, and GO terms or KEGG pathways with an FDR‐adjusted *q* value < 0.05 were considered statistically significant.

### 2.5 Statistical Analysis

Statistical analysis was performed using SPSS Statistics 25.0 software (IBM Corp., NY, USA), R v4.5.1 (R Core team), and R Studio 2023.03.0 (Posit Software). The normality of data distribution was assessed with the one‐sample Kolmogorov–Smirnov test. Normally distributed quantitative data are presented as “mean ± standard deviation”, and non‐normally distributed data as “median (interquartile range).” Differences between groups were evaluated using the nonparametric test or the student *t*‐test. Percentages were compared using the chi‐square test. For cross‐sectional studies, Firth‐penalized logistic regression was employed to calculate intergroup differences while mitigating the effects of extreme values or small subgroup sizes. For cohort studies, Cox survival analysis was used to compare intergroup differences, with Kaplan–Meier survival analysis additionally applied to estimate disparities among groups. Among them, *p* ≤ 0.05 means statistical significance. For clinical statistical analyses, a two‐sided *p*‐value ≤ 0.05 was considered statistically significant. For serum antibody omics and enrichment analyses, statistical significance was determined using FDR‐adjusted q values after Benjamini–Hochberg correction, with *q* < 0.05 considered significant.

## 3 Results

### 3.1 The European SHARE Database

Participants were divided into groups based on the presence or absence of “Diabetes,” and intergroup differences in gender, age, height, weight, BMI, left and right handgrip strength, medication history, smoking and alcohol history, osteoporosis, fractures, and falls were analyzed. Except for smoking history (*p* = 0.253), all other variables exhibited statistically significant differences (all *p* < 0.001). The data showed that males accounted for 43.6% (142 643 cases), with a median age of 66 years. Individuals with diabetes constituted 14.75% (48 276 cases) of the total population. (As shown in Supplementary Table 1).

To elucidate the relationships between the events (osteoporosis, falls, fractures) and the variables (diabetes, grip strength), a binary logistic regression analysis with Firth penalization was performed. After adjusting for gender, age, BMI, medication history, and smoking/alcohol history, individuals with diabetes were more likely to develop osteoporosis, falls, and fractures compared to those without diabetes (OP: *p* < 0.001, OR 1.194, 95% CI 1.102–1.292; falls: *p* < 0.001, OR 1.351, 95% CI 1.273–1.433; fractures: *p* < 0.001, OR 1.237, 95% CI 1.143–1.336). Furthermore, an inverse relationship was observed between both left and right handgrip strength and the incidence of osteoporosis, falls, and fractures (Left Grip: OP: *p* < 0.001, OR 0.983, 95% CI 0.979–0.987; falls: *p* < 0.001, OR 0.987, 95% CI 0.984–0.990; fractures: *p* = 0.372, OR 1.002, 95% CI 0.998–1.006. Right Grip: OP: *p* < 0.001, OR 0.989, 95% CI 0.985–0.993; falls: *p* < 0.001, OR 0.979, 95% CI 0.976–0.982; fractures: *p* < 0.001, OR 0.990, 95% CI 0.986–0.994). (As shown in Figure 1).

**FIGURE 1:**
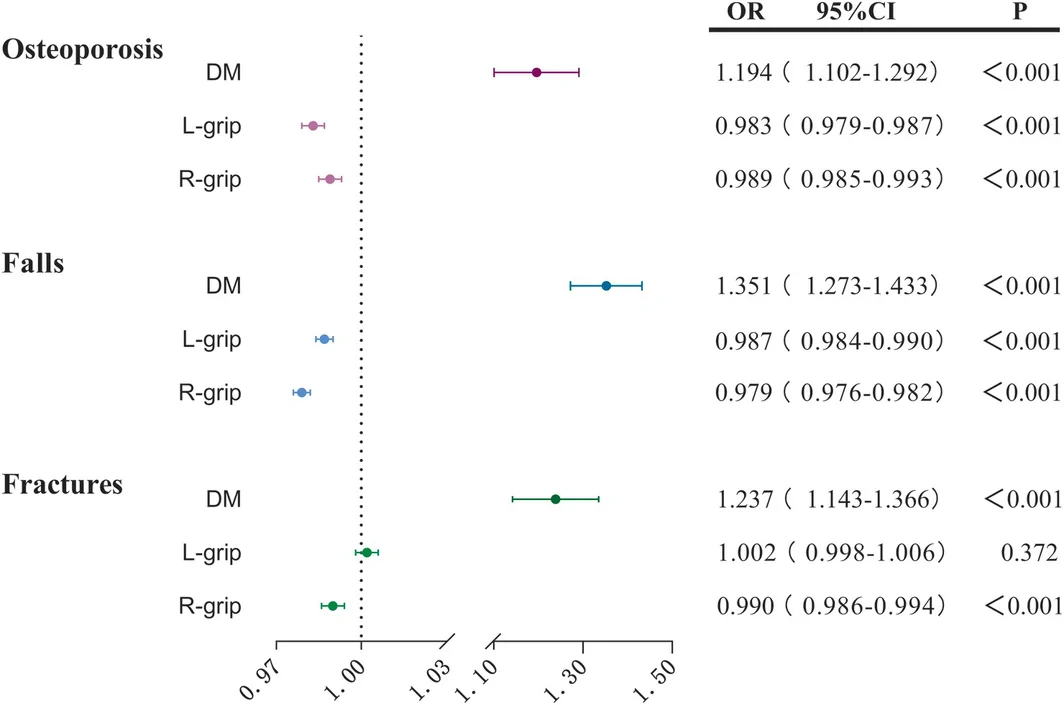
Forest plot of the binary logistic regression analysis from the European SHARE database. L‐grip = Left handgrip strength; R‐grip = Right handgrip strength.

### 3.2 Local Database

The local database comprised 6637 cases, with 53.1% being male (3327 cases), and a median age of 62 years. Based on the presence of DPN and/or LAMM, the study population was categorized into four groups: the non‐DPN nonsarcopenia group (None) with 3443 cases; the LAMM‐only group (LAMM) with 1927 cases; the DPN‐only (DPN) group with 794 cases; and the comorbid LAMM and DPN group (LAMM + DPN) with 473 cases. The study revealed a statistically significant gradual increase in the detection rates of osteoporosis, falls, and fractures as the condition progressed from the None group to the SP&DPN group (*p* < 0.001). A similar trend was observed in the risks of fractures and falls (Both *p* < 0.001) (as shown in Table 1). To enhance data visualization, frequency distribution chart (as shown in Figure 2a) and heat map of positive events (as shown in Figure 2b) were plotted.

**TABLE 1:** Baseline characteristics table of the local database.

Variable	Total (6637)	None (3443)	LAMM (1927)	DPN (794)	LAMM + DPN (473)	*p*
Age (years), median (IQR)	62.00 (53.00–69.00)	60.00 (51.00–68.00)	63.00 (54.00–71.00)	63.00 (56.00–70.00)	66.00 (57.00–74.00)	< 0.001
Male, *n* (%)	3527 (53.1)	1654 (48.0)	1231 (63.9)	316 (39.8)	326 (68.9)	< 0.001
Course of DM (years), median (IQR)	8.00 (3.00–13.00)	7.00 (2.00–12.00)	8.00 (2.00–12.00)	10.00 (6.00–16.00)	10.00 (5.00–15.00)	< 0.001
BMI (kg/m^2^), median (IQR)	24.69 (22.43–27.18)	25.56 (23.92–27.70)	22.58 (20.70–24.69)	26.05 (24.00–28.14)	22.04 (20.28–24.03)	< 0.001
WHtR, median (IQR)	0.54 (0.51–0.58)	0.55 (0.52–0.59)	0.51 (0.48–0.55)	0.57 (0.53–0.61)	0.52 (0.48–0.55)	< 0.001
BF, median (IQR)	0.29 (0.23–0.34)	0.30 (0.25–0.36)	0.26 (0.20–0.32)	0.31 (0.23–0.36)	0.24 (0.18–0.30)	< 0.001
QTc (s), median (IQR)	0.42 (0.40–0.44)	0.42 (0.40–0.44)	0.42 (0.40–0.44)	0.42 (0.40–0.44)	0.42 (0.40–0.43)	< 0.001
SBP (mmHg), median (IQR)	134.00 (122.00–148.00)	134.00 (123.00–147.00)	130.00 (120.00–144.00)	140.00 (127.00–155.75)	138.00 (126.00–155.00)	< 0.001
DBP (mmHg), median (IQR)	78.00 (72.00–85.00)	79.00 (73.00–86.00)	78.00 (70.00–84.00)	79.00 (71.00–86.00)	78.00 (70.00–84.00)	< 0.001
HT, *n* (%)	4298 (64.8)	2218 (64.4)	1163 (60.4)	572 (72.0)	345 (72.9)	< 0.001
Smoking, *n* (%)	1405 (21.2)	667 (19.4)	468 (24.3)	119 (15.0)	151 (31.9)	< 0.001
Drinking, *n* (%)	564 (8.5)	277 (8.0)	164 (8.5)	53 (6.7)	70 (14.8)	< 0.001
Metformin, *n* (%)	3644 (54.9)	1805 (52.4)	1098 (57.0)	479 (60.3)	262 (55.4)	< 0.001
Thiazolidinedione, *n* (%)	566 (8.5)	279 (8.1)	168 (8.7)	77 (9.7)	42 (8.9)	0.502
SGLT2i, *n* (%)	464 (7.0)	299 (8.7)	159 (8.3)	4 (0.5)	2 (0.4)	< 0.001
DPP4i, *n* (%)	1081 (16.3)	654 (19.0)	373 (19.4)	32 (4.0)	22 (4.7)	< 0.001
GLP1ra, *n* (%)	958 (14.4)	609 (17.7)	339 (17.6)	7 (0.9)	3 (0.6)	< 0.001
Other hypoglycemic drugs, *n* (%)	4402 (66.3)	2225 (64.6)	1248 (64.8)	580 (73.0)	349 (73.8)	< 0.001
Insulin, *n* (%)	3316 (50.0)	1559 (45.3)	958 (49.7)	501 (63.1)	298 (63.0)	< 0.001
ACEI/ARB, *n* (%)	1585 (23.9)	838 (24.3)	429 (22.3)	188 (23.7)	130 (27.5)	0.087
Beta blocker, *n* (%)	593 (8.9)	314 (9.1)	182 (9.4)	51 (6.4)	46 (9.7)	0.063
CCB, *n* (%)	1761 (26.5)	915 (26.6)	476 (24.7)	232 (29.2)	138 (29.2)	0.047
Diuretic, *n* (%)	339 (5.1)	178 (5.2)	82 (4.3)	52 (6.5)	27 (5.7)	0.083
Statins, *n* (%)	1697 (25.6)	1019 (29.6)	527 (27.3)	92 (11.6)	59 (12.5)	< 0.001
GLU (mmol/L), median (IQR)	7.77 (5.82–10.83)	7.77 (5.87–10.61)	7.87 (5.87–11.34)	7.79 (5.63–10.94)	7.49 (5.34–10.64)	0.011
HbA1c (%), median (IQR)	8.80 (7.20–10.70)	8.70 (7.10–10.50)	8.90 (7.30–11.00)	8.80 (7.30–10.50)	9.00 (7.20–11.00)	0.002
ALB (g/L), median (IQR)	39.97 (37.00–42.70)	40.70 (37.99–43.20)	39.35 (36.30–42.20)	39.30 (36.20–41.90)	38.00 (34.90–40.90)	< 0.001
TC (mmol/L), median (IQR)	4.50 (3.74–5.33)	4.55 (3.81–5.36)	4.40 (3.65–5.19)	4.60 (3.81–5.48)	4.32 (3.60–5.26)	< 0.001
TG (mmol/L), median (IQR)	1.45 (1.01–2.18)	1.58 (1.10–2.30)	1.28 (0.92–1.93)	1.48 (1.01–2.30)	1.19 (0.82–1.82)	< 0.001
CR (μmol/L), median (IQR)	60.90 (49.40–75.60)	60.70 (49.50–74.60)	60.00 (48.90–74.70)	62.85 (50.45–79.00)	64.30 (50.00–81.00)	< 0.001
eGFR (mL/min), median (IQR)	97.89 (85.71–108.64)	99.07 (87.37–109.23)	98.23 (87.04–109.84)	94.09 (76.50–104.05)	93.80 (78.52–106.75)	< 0.001
Na (mmol/L), median (IQR)	141.20 (139.30–143.00)	141.30 (139.50–143.00)	141.00 (139.00–143.00)	141.60 (139.46–143.00)	141.00 (139.00–143.00)	0.008
K (mmol/L), median (IQR)	4.02 (3.73–4.31)	4.03 (3.76–4.32)	3.99 (3.69–4.29)	4.02 (3.71–4.36)	4.02 (3.68–4.32)	0.001
Ca (mmol/L), median (IQR)	2.23 (2.15–2.32)	2.24 (2.16–2.33)	2.23 (2.14–2.32)	2.22 (2.12–2.31)	2.21 (2.12–2.30)	< 0.001
VD (ng/mL), median (IQR)	22.88 (19.46–25.67)	22.92 (20.29–26.36)	22.90 (19.54–26.63)	22.76 (17.86–23.28)	22.56 (16.53–23.33)	< 0.001
Total PINP (ng/mL), median (IQR)	41.59 (30.62–44.28)	41.59 (31.16–45.17)	40.89 (28.81–43.90)	42.16 (33.46–43.57)	40.91 (30.03–43.25)	< 0.001
CTX (ng/mL), median (IQR)	0.47 (0.28–0.68)	0.45 (0.27–0.66)	0.49 (0.29–0.69)	0.46 (0.27–0.69)	0.47 (0.27–0.69)	0.008
UACR (mg/g), median (IQR)	15.92 (7.10–68.88)	14.50 (6.64–51.59)	14.53 (6.92–60.23)	26.19 (8.92–216.53)	29.78 (10.55–203.61)	< 0.001
DF, *n* (%)	249 (3.8)	83 (2.4)	56 (2.9)	54 (6.8)	56 (11.8)	< 0.001
DKD, *n* (%)	2589 (39.0)	1213 (35.2)	728 (37.8)	402 (50.6)	246 (52.0)	< 0.001
DR, *n* (%)	1491 (22.5)	726 (21.1)	361 (18.7)	259 (32.6)	145 (30.7)	< 0.001
OP, *n* (%)	1230 (18.5)	485 (14.1)	439 (22.8)	168 (21.2)	138 (29.2)	< 0.001
Falls, *n* (%)	509 (7.7)	156 (4.5)	202 (10.5)	58 (7.3)	93 (19.7)	< 0.001
High risk of falls, *n* (%)	460 (6.9)	129 (3.7)	197 (10.2)	52 (6.5)	82 (17.3)	< 0.001
Fractures, *n* (%)	424 (6.4)	163 (4.7)	141 (7.3)	74 (9.3)	46 (9.7)	< 0.001
High risk of fractures, *n* (%)	1931 (29.1)	628 (18.2)	868 (45.0)	207 (26.1)	228 (48.2)	< 0.001

*Note:* “None” refers to the patient group without diabetic peripheral neuropathy and without low appendicular muscle mass.; “LAMM” refers to the patient group with only low appendicular muscle mass; “DPN” refers to the group of patients with only clinically diagnosed diabetic peripheral neuropathy; “LAMM + DPN” means the comorbid low appendicular muscle mass and clinically diagnosed diabetic peripheral neuropathy group. *p* ≤ 0.05 means statistical significance.

Abbreviations: ACEI/ARB, angiotensin‐converting enzyme inhibitors or angiotensin II receptor blockers; ALB, albumin; BF, body fat ratio; BMI, body mass index; Ca, calcium; CCB, calcium channel blockers; Course of DM, course of diabetes mellitus; CR, creatinine; CTX, Type I collagen cross‐linked C‐telopeptide; DBP, diastolic blood pressure; DF, diabetic foot; DKD, diabetic kidney disease; DPP4i, dipeptidyl peptidase‐4 inhibitors; DR, diabetic retinopathy; eGFR, estimated glomerular filtration rate; GLP1ra, glucagon‐like peptide‐1 receptor agonists; GLU, glucose; HbA1c, glycated hemoglobin A1c; HT, hypertension; K, kalium; Na, natrium; OP, osteoporosis; QTc, corrected QT interval; SBP, systolic blood pressure; SGLT2i, sodium‐glucose linked transporter 2 inhibitors; TC, total cholesterol; TG, triglycerides; Total PINP, Type I procollagen N‐terminal propeptide; UACR, urinary albumin‐to‐creatinine ratio; VD, 25‐hydroxyvitamin D; WHtR, waist‐to‐height ratio.

**FIGURE 2:**
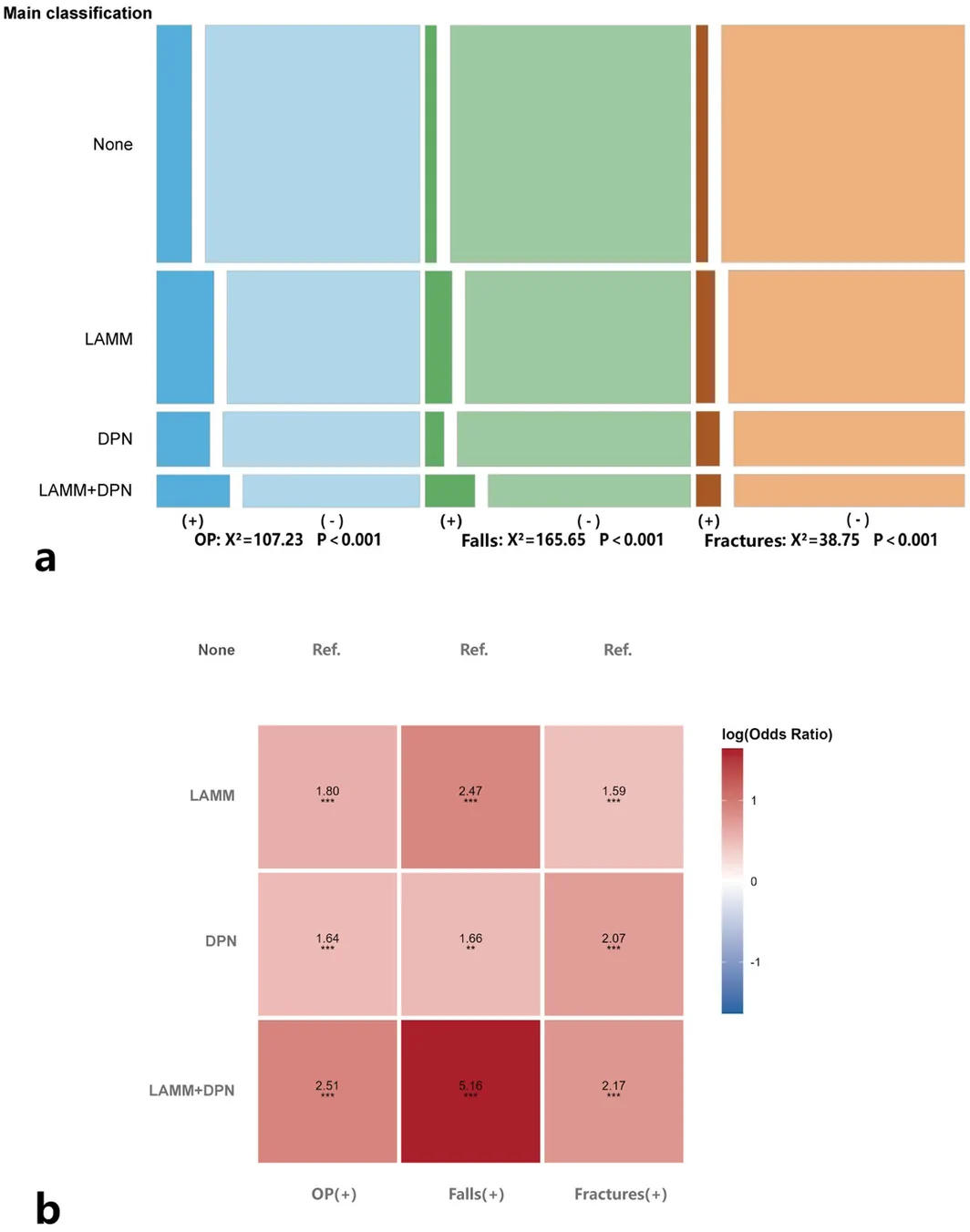
Frequency distribution charts (a) and heat maps of positive events (b) of Local Database. Main classification includes: “None” refers to the patient group without diabetic peripheral neuropathy and without low appendicular muscle mass. “LAMM” refers to the patient group with only low appendicular muscle mass; “DPN” refers to the group of patients with only clinically diagnosed diabetic peripheral neuropathy; “LAMM + DPN” means the comorbid low appendicular muscle mass and clinically diagnosed diabetic peripheral neuropathy group. *p* ≤ 0.05 means statistical significance.

To elucidate the relationship between osteoporosis, falls, and fractures across different patient groups, potential associations were analyzed using Firth‐penalized binary logistic regression. After adjusting for age, duration of diabetes, BMI, WHtR, BF, UACR, TC, TG, ALB, eGFR, HbA1c, VD, total PINP, CTX, gender, DR, DKD, DF, smoking and medication (including metformin, thiazolidinediones, SGLT2i, DPP4i, GLP1ra, other hypoglycemic drugs), as well as history of hypertension, ACEI/ARB, and statins, the results demonstrated that compared to the None group, another three groups all exhibited significantly higher risks of osteoporosis, falls, and fractures, with the LAMM + DPN group consistently showing the highest risk across all outcomes (LAMM + DPN: osteoporosis: *p* < 0.001, OR 2.151, 95% CI 1.645–2.805; falls: *p* < 0.001, OR 4.667, 95% CI 3.406–6.376; fractures: *p* < 0.001, OR 2.416, 95% CI 1.367–3.515). (As shown in Supplementary Table 2 and Figure 3).

**FIGURE 3:**
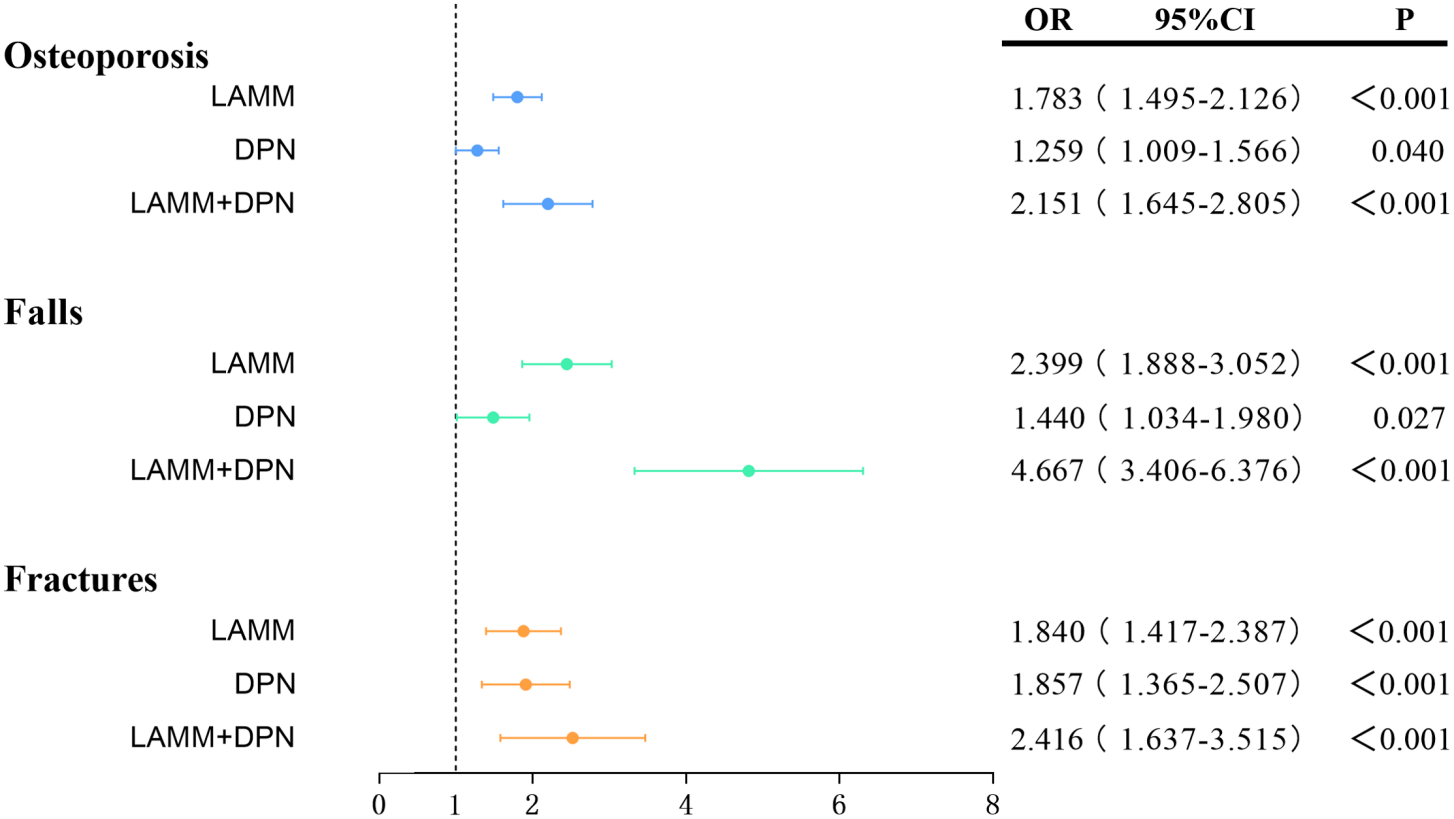
Forest plot of the firth‐penalized binary logistic regression analysis from local database. “LAMM” refers to the patient group with only low appendicular muscle mass; “DPN” refers to the group of patients with only clinically diagnosed diabetic peripheral neuropathy; “LAMM + DPN” means the comorbid low appendicular muscle mass and clinically diagnosed diabetic peripheral neuropathy group. The reference group within the group is based on the level of “None”. *p* ≤ 0.05 is significantly significant. Adjusted for the following variables: Age, course of DM, BMI, WHtR, BF, UACR, TC, TG, ALB, eGFR, HbA1c, VD, total PINP, CTX, gender, DR, DKD, DF, metformin, other hypoglycemic drugs, thiazolidinedione, SGLT2i, DPP4i, GLP1ra, ACEI/ARB, statins, smoking, HT.

Subsequently, Cox proportional hazards regression analyses were performed. After adjusting for age, duration of diabetes, BMI, WHtR, BF, UACR, TC, TG, ALB, eGFR, HbA1c, VD, total PINP, CTX, gender, DR, DKD, DF, smoking and medication (including metformin, thiazolidinediones, SGLT2i, DPP4i, GLP1ra, other hypoglycemic drugs), as well as history of hypertension, ACEI/ARB, and statins, we performed COX survival analysis and plotted survival‐curves for the three outcome events. The results showed that the LAMM + DPN group exhibited the most rapid decline in survival curves, indicating the highest risk of osteoporosis and falls (LAMM + DPN: osteoporosis: *p* = 0.002, HR 3.410, 95% CI 1.579–7.362; falls: *p* < 0.001, HR 4.385, 95% CI 1.767–9.130), however, in terms of fractures, no significant differences were observed among all groups (Both *p* > 0.05). (As shown in Table 2, Figure 4 and Supplementary Table 3) Additionally, we analyzed data from a subpopulation without the outcome events at baseline, following the same analytical steps to examine differences among the four groups. Only some groups showed statistically significant differences for specific outcome events (LAMM: falls: *p* = 0.020, HR 2.571, 95% CI 1.154–5.494). (As shown in Supplementary Table 4 and Supplementary Figure 1).

**TABLE 2:** Results from the COX survival analysis of the local database.

Dependent variables	Independent variables	HR	95% CI_low	95% CI_high	*p*
OP	None	1.000	Ref.	Ref.	—
	LAMM	1.285	0.760	2.171	0.349
	DPN	0.856	0.482	1.521	0.597
	LAMM + DPN	3.410	1.579	7.362	0.002
Falls	None	1.000	Ref.	Ref.	—
	LAMM	2.690	1.528	4.735	< 0.001
	DPN	1.163	0.564	2.399	0.683
	LAMM + DPN	4.385	1.838	10.459	< 0.001
Fractures	None	1.000	Ref.	Ref.	—
	LAMM	1.151	0.599	2.213	0.673
	DPN	0.772	0.355	1.679	0.514
	LAMM + DPN	0.708	0.187	2.675	0.611

*Note:* Patients were not subjected to any selection or intervention at baseline. “None” refers to the patient group without diabetic peripheral neuropathy and without low appendicular muscle mass.; “LAMM” refers to the patient group with only low appendicular muscle mass; “DPN” refers to the group of patients with only clinically diagnosed diabetic peripheral neuropathy; “LAMM + DPN” means the comorbid low appendicular muscle mass and clinically diagnosed diabetic peripheral neuropathy group. The reference group within the group is based on the level of “None”. *p* ≤ 0.05 is significantly significant. Adjusted for the following variables: age, course of DM, BMI, WHtR, BF, UACR, TC, TG, ALB, eGFR, HbA1c, VD, total PINP, CTX, gender, DR, DKD, DF, metformin, other hypoglycemic drugs, thiazolidinedione, SGLT2i, DPP4i, GLP1ra, ACEI/ARB, statins, smoking, HT.

Abbreviations: CI, confidence interval; HR, hazard ratio; OP, Osteoporosis.

**FIGURE 4:**
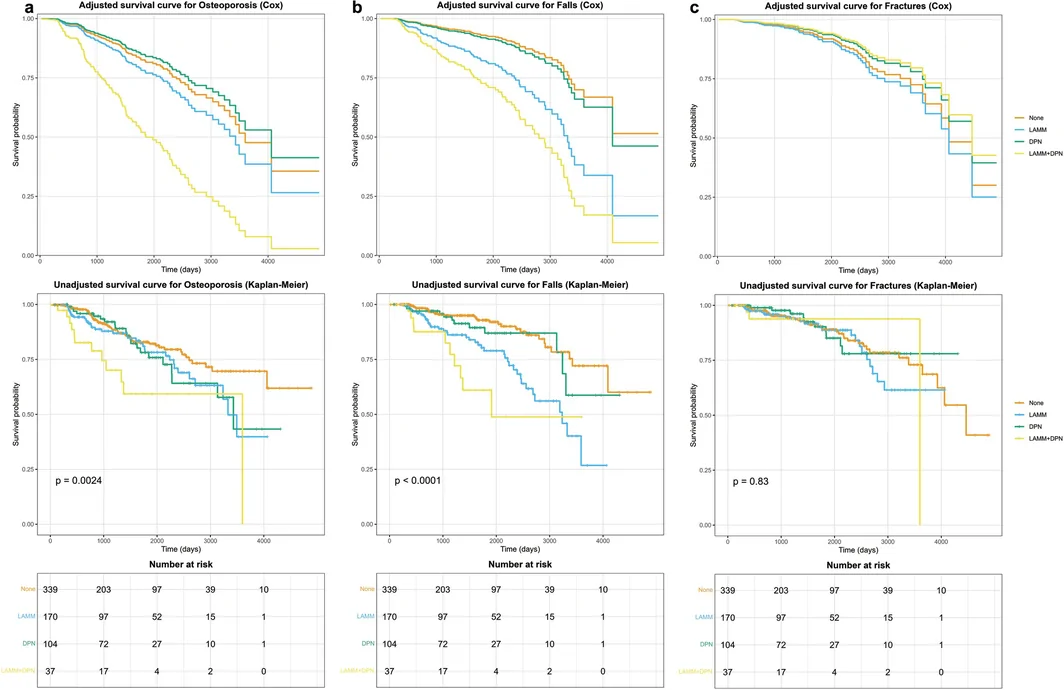
Cumulative survival curves and frequency table from COX and Kaplan–Meier analyses of the local database. From left to right: (a) Survival analysis curve and frequency table for outcome event OP; (b) Survival analysis curve and frequency table for outcome event Falls; (c) Survival analysis curve and frequency table for outcome event Fractures. The reference group within the group is based on the level of “None”. *p* ≤ 0.05 is significantly significant. Adjusted for the following variables: Age, course of DM, BMI, WHtR, BF, UACR, TC, TG, ALB, eGFR, HbA1c, VD, total PINP, CTX, gender, DR, DKD, DF, metformin, other hypoglycemic drugs, thiazolidinedione, SGLT2i, DPP4i, GLP1ra, ACEI/ARB, statins, smoking, HT. The orange line corresponds to the none group, the blue line to the LAMM group, the green line to the DPN group, and the yellow line to the LAMM + DPN group.

### 3.3 Serum Antibody Omics

To further explore potential biological links between coexisting DPN and LAMM and adverse skeletal outcomes, serum antibody omics analysis was performed in 182 patients with both clinical data and serum samples available. Patients were classified into four groups according to the presence or absence of DPN and LAMM: None, LAMM, DPN, and LAMM + DPN.

After prespecified low‐count filtering, peptides with a count greater than 1 in at least 10% of the samples (≥ 19 of 182 samples) were retained. A total of 854 peptides met this criterion and were included in the subsequent differential antibody reactivity analysis. To control for false positives caused by multiple comparisons, the Benjamini–Hochberg procedure was applied for false discovery rate correction. Using an FDR‐adjusted *q* value < 0.05 and an absolute fold change > 2 as the criteria for differentially reactive peptides, FDR‐significant peptides were identified only in the LAMM + DPN group compared with the None group. Specifically, 9 differentially reactive peptides reached statistical significance after FDR correction, corresponding to 8 representative gene/protein annotations, including FOXP2, INAVA, UFD1, CIC, PRDM15, SLC43A2, ZMAT5, and NP_001354759.1. In contrast, no FDR‐significant differentially reactive peptides were identified in either the LAMM group or the DPN group compared with the None group.

Among these candidate molecules, FOXP2 and INAVA showed the strongest statistical signals. KEGG enrichment analysis showed that the protein processing in endoplasmic reticulum pathway and the spinocerebellar ataxia pathway remained significant after FDR correction. GO enrichment analysis did not identify any terms that remained significant after FDR correction.

To contextualize the exploratory enrichment signals, Figure 5 presents a literature‐informed hypothetical network, not a causal model. The literature links endoplasmic reticulum dysfunction to protein misfolding and endoplasmic reticulum stress, which has been implicated in DPN [23], DPN is associated with sarcopenia‐related muscle loss and osteoporosis [11, 12], while endoplasmic reticulum stress may contribute to both conditions [24], which may influence each other [25]. Endoplasmic reticulum stress has also been implicated in spinocerebellar ataxia, either directly [26] or through a proposed sequence involving INAVA‐amplified inflammation, inflammatory bowel disease, and gut‐brain‐axis effects on the central nervous system [27, 28]. Sarcopenia‐related muscle loss, osteoporosis, and spinocerebellar ataxia are each associated with falls and fractures [9, 29] (as shown in Figure 5). These connections are literature‐supported hypotheses prompted by the enrichment signals, not evidence of causal mediation in our cohort.

**FIGURE 5:**
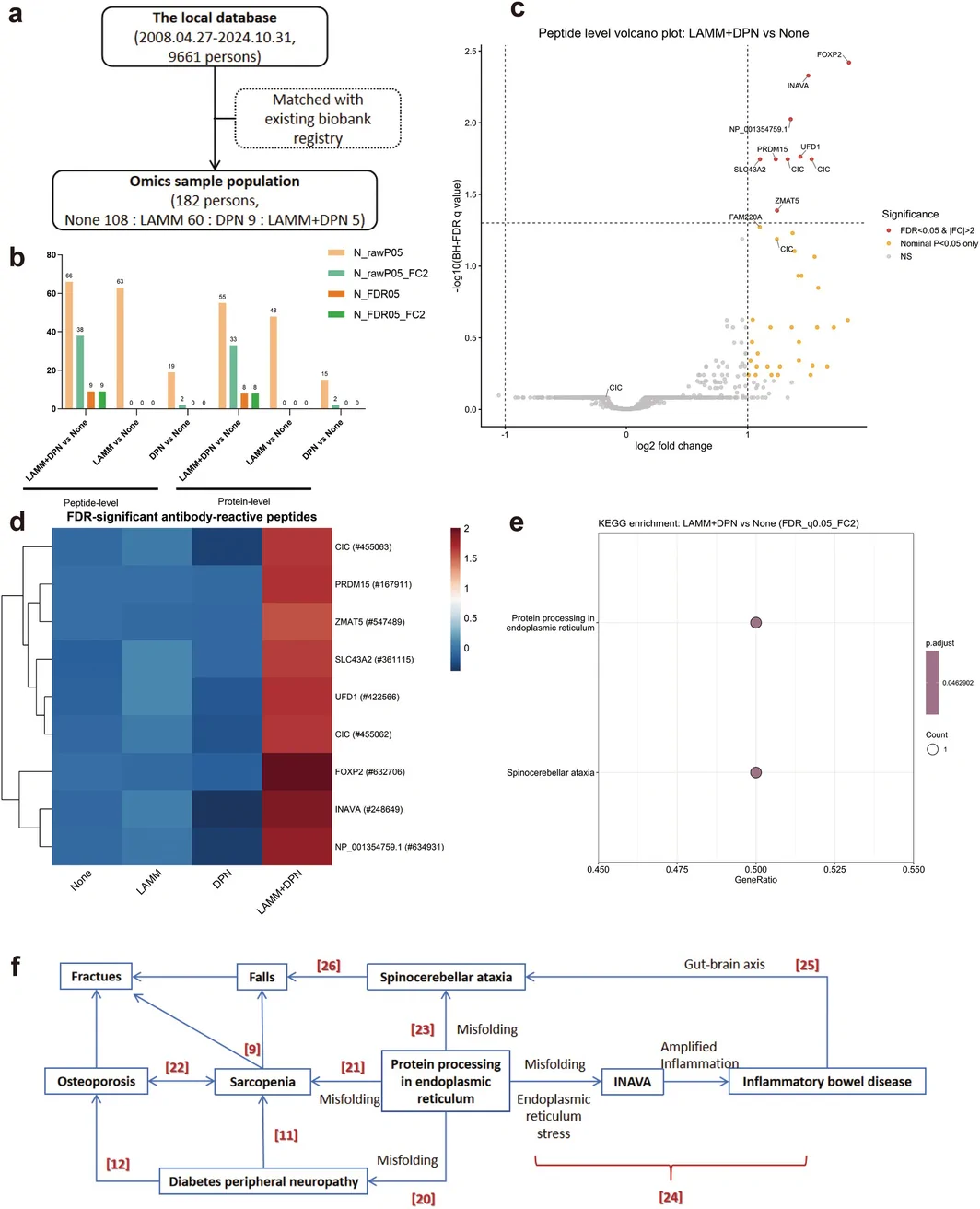
Serum antibody omics analysis and exploratory mechanistic network. (a) Flow diagram of sample selection for serum antibody omics analysis. A total of 182 patients with matched clinical data and available serum samples were classified into the None, LAMM, DPN, and LAMM + DPN groups. (b) Summary of differentially reactive features across group comparisons at the peptide level and representative gene/protein annotation level. FDR‐significant features were identified only in the LAMM + DPN versus None comparison. (c) Peptide‐level volcano plot for the LAMM + DPN versus None comparison. Red points indicate peptides with FDR‐adjusted *q* < 0.05 and absolute fold change > 2. (d) Heatmap of the 9 FDR‐significant antibody‐reactive peptides across the four clinical groups. Values are shown as row‐scaled z scores of logCPM‐normalized peptide counts. (e) KEGG enrichment analysis of representative gene/protein annotations from the LAMM + DPN versus None comparison, showing significant enrichment in protein processing in the endoplasmic reticulum and spinocerebellar ataxia pathways after FDR correction. (f) Proposed exploratory mechanistic network linking diabetic peripheral neuropathy, sarcopenia, osteoporosis, falls, and fractures. LAMM, low appendicular muscle mass; DPN, clinically diagnosed diabetic peripheral neuropathy; LAMM + DPN, low appendicular muscle mass combined with diabetic peripheral neuropathy; None, patients without low appendicular muscle mass or diabetic peripheral neuropathy; FDR, false discovery rate; BH, Benjamini–Hochberg; FC, fold change; logCPM, log_2_ counts per million; KEGG, Kyoto Encyclopedia of Genes and Genomes; ER, endoplasmic reticulum.

Overall, patients with coexisting DPN and LAMM may have an antibody reactivity profile distinct from patients with either condition alone. The enriched pathways offer preliminary clues involving protein processing, immune responses, and neurodegeneration, but do not establish mediation of the clinical associations. Given the limited subgroup sizes, independent replication and functional validation are required.

## 4 Discussion

DPN is a form of diabetic microangiopathy involving multiple mechanisms and signaling pathways, including enhanced polyol metabolism, accumulation of advanced glycation end products, pro‐inflammatory responses, and oxidative stress [30, 31, 32]. Concurrently, oxidative stress serves as a significant pathway contributing to the pathogenesis of sarcopenia [33, 34]. More broadly, osteoporosis is not solely an age‐related disorder but is also associated with chronic inflammation and oxidative stress [35, 36]. As conditions sharing overlapping mechanistic pathways, the interrelationships among DPN, sarcopenia, and osteoporosis have attracted considerable scholarly attention, prompting ongoing exploration and research into their common pathophysiological foundations.

Our study investigated the combined impact of DPN and LAMM on osteoporosis, and subsequently evaluated the incidence of falls and fractures. We initially conducted a pilot study using a public database to explore the effects of diabetes and grip strength on osteoporosis, falls, and fractures. The results indicated that individuals with diabetes are more prone to osteoporosis, falls, and fractures, and a similar trend was observed in those with reduced grip strength. As the public database lacks specific variables for DPN and sarcopenia, grip strength was used as a surrogate measure [6]. A similar pattern was observed in the database of the First Affiliated Hospital of Fujian Medical University: coexisting DPN and LAMM was associated with higher risks of osteoporosis, falls, and fractures. These findings are consistent with previous research: Hui Chen et al. reported significant bone mineral metabolism disorders in patients with Type 2 diabetes, involving both bone resorption and formation processes [37]. Alec T. Beeve et al. proposed that DPN may affect skeletal health through three potential pathways: neurotrauma, neurotrophic effects, and neurovascular mechanisms [38]. Sarodnik, C. et al. suggested that neuropathy leads to lower limb muscle atrophy and weakness, and the presence of sarcopenia increases the risk of falls, thereby elevating the likelihood of fractures [39]. Furthermore, the coexistence of sarcopenia and osteoporosis further amplifies the risk of falls and fractures [40].

Figure 5 integrates the FDR‐significant enrichment signals with literature‐reported relationships into a provisional, hypothesis‐generating network. The network was not directly tested in our cohort and should not be interpreted as a validated mechanism. It serves only to contextualize the omics findings and prioritize pathways for future targeted experiments.

The strengths of this study are multifaceted. We innovatively integrate DPN with LAMM, moving beyond single‐disease investigations. The large clinical database and extended follow‐up enhance the representativeness of our findings for natural populations. Using a public database for preliminary analysis guided subsequent research, while immunopeptidomics provides mechanistic clues for potential subsequent research.

This study has several limitations. First, the local cohort consisted of hospitalized patients, who may have more severe metabolic abnormalities, a higher complication burden, and more complex comorbidities than community‐dwelling patients with diabetes. Therefore, selection bias may exist, and the findings should not be directly generalized to the broader community‐based diabetes population. Second, ethnic and population differences between the European SHARE database and the Chinese local cohort should be considered. In addition, grip strength in SHARE was used only as a surrogate marker of muscle function and cannot fully represent DXA‐defined low appendicular muscle mass. Therefore, the SHARE analysis should be interpreted as exploratory population‐level support rather than external validation. Third, although serum antibody profiling suggested potential involvement of endoplasmic reticulum protein processing, no functional experiments or validation of classical endoplasmic reticulum stress markers were performed. Thus, the omics findings should be regarded as exploratory mechanistic clues requiring further validation.

This study explored the interrelationships among DPN, LAMM, and osteoporosis, providing preliminary biological clues regarding potential links among these conditions. These findings highlight the importance of disease coexistence and encourage clinicians to adopt a multifaceted approach rather than focusing on isolated diseases.

## 5 Conclusion

In patients with Type 2 diabetes, coexisting clinically diagnosed DPN and LAMM were associated with higher odds of osteoporosis, falls, and fractures and with higher longitudinal hazards of osteoporosis and falls, but not fractures. Exploratory serum antibody omics provided preliminary, hypothesis‐generating clues involving endoplasmic reticulum protein processing, immune‐response pathways, and neurodegeneration‐related pathways.

## Author Contributions

**Biao Zheng:** investigation, methodology, software, validation, visualization, writing – original draft. **Yongze Zhang:** investigation, methodology, software, validation, visualization, writing – original draft. **Dan Liang:** methodology, software, visualization. **Ximei Shen:** validation, writing – review and editing. **Lingning Huang:** validation, writing – review and editing. **Fengying Zhao:** data curation. **Sunjie Yan:** conceptualization, data curation, funding acquisition, supervision, writing – review and editing.

## Funding

This study was supported by the following projects: Scientific and Technological Major Special Project of Fujian Provincial Health Commission (No. 2021ZD01004); Research on the Prevention and Treatment of Cancer, Cardiovascular and Cerebrovascular Diseases, Respiratory Diseases, and Metabolic Diseases National Key Technologies R&D Program (No. 2023ZD0509005); and National Natural Science Foundation of China (grant number No. 82371601).

## Conflicts of Interest

The authors declare no conflicts of interest.

## Supporting information


**Supplementary Figure 1:** Cumulative survival curves and frequency table from COX and Kaplan–Meier analyses of the local database.

**Supplementary Figure 2:** *The local database means the Diabetic Database of the Department of Endocrinology, The First Affiliated Hospital of Fujian Medical University. # “None” refers to the patient group without diabetic peripheral neuropathy and without low appendicular muscle mass.; “LAMM” refers to the patient group with only low appendicular muscle mass; “DPN” refers to the group of patients with only clinically diagnosed diabetic peripheral neuropathy; “LAMM + DPN” means the comorbid low appendicular muscle mass and clinically diagnosed diabetic peripheral neuropathy group.

**Supplementary Table 1:** Baseline characteristics table of the European SHARE database.

**Supplementary Table 2:** Results from the firth‐penalized binary logistic regression analysis of the local database.

**Supplementary Table 3:** Baseline and follow‐up demographic characteristics of 651 patients.

**Supplementary Table 4:** Results from the COX survival analysis of the local database.

**Supplementary Tables 5:** Significant serum antibody‐reactive peptides and representative annotations.
**Supplementary Table 5a:** Differentially reactive peptides identified in the LAMM + DPN versus none comparison.
**Supplementary Table 5b:** Representative gene/protein annotations corresponding to the significant peptides in the LAMM + DPN versus none comparison.

## Data Availability

The public data underlying this article are available in [the European SHARE database], at https://doi.org/10.6103/SHARE.w8.900. The database is derived from sources in the public domain: [https://share‐eric.eu/]. The local database for this study is not publicly available because of concerns regarding participant/patient anonymity. Requests to access the database should be directed to the corresponding authors.
