# Supplementary material for: Diabetic Peripheral Neuropathy With Low Appendicular Muscle Mass Is Associated With Osteoporosis, Falls, and Fractures
Source: J Diabetes. 2026 Oct 28;18(11):e70276. doi: 10.1111/1753-0407.70276 (PMC13715079; doi:10.1111/1753-0407.70276)
Supplement: Supplementary file 1 [file JDB-18-e70276-s001.docx]

**Supplementary Table 1:**

**Baseline Characteristics Table of the European SHARE Database.**

| **Variable** | **Total (327248)** | **Non-Diabetes (278972)** | **Diabetes (48276)** | **P value** |
| --- | --- | --- | --- | --- |
| Male, n (%) | 142643 (43.6) | 119536 (42.8) | 23107 (47.9) | ＜0.001 |
| Age (years), median (IQR) | 66.00 (59.00-74.00) | 65.00 (58.00-73.00) | 70.00 (63.00-77.00) | ＜0.001 |
| Height (m), median (IQR) | 1.68 (1.62-1.74) | 1.68 (1.62-1.74) | 1.68 (1.60-1.73) | ＜0.001 |
| Weight (kg), median (IQR) | 75.00 (65.00-85.00) | 75.00 (65.00-84.00) | 80.00 (70.00-90.00) | ＜0.001 |
| BMI (kg/m^2^), median (IQR) | 26.45 (23.88-29.30) | 26.12 (23.63-28.73) | 28.09 (25.62-31.65) | ＜0.001 |
| L-grip, median (IQR) | 30.00 (23.00-37.00) | 30.00 (23.00-37.00) | 30.00 (21.00-34.00) | ＜0.001 |
| R-grip, median (IQR) | 32.65 (25.00-40.00) | 32.65 (25.00-40.00) | 32.00 (23.00-37.00) | ＜0.001 |
| DM drugs, n (%) | 38354 (11.7) | 1544 (0.6) | 36810 (76.2) | ＜0.001 |
| HT drugs, n (%) | 137188 (41.9) | 104927 (37.6) | 32261 (66.8) | ＜0.001 |
| Drinking, n (%) | 218429 (66.7) | 192399 (69.0) | 26030 (53.9) | ＜0.001 |
| Smoking, n (%) | 148995 (45.5) | 127131 (45.6) | 21864 (45.3) | 0.253 |
| HT, n (%) | 151091 (46.2) | 115983 (41.6) | 35108 (72.7) | ＜0.001 |
| Falls, n (%) | 21837 (6.7) | 16308 (5.8) | 5529 (11.5) | ＜0.001 |
| Fractures, n (%) | 11933 (3.6) | 9404 (3.4) | 2529 (5.2) | ＜0.001 |
| OP, n (%) | 13776 (4.2) | 11531 (4.1) | 2245 (4.7) | ＜0.001 |

^Notes: BMI = Body mass index; L-grip = Left handgrip strength; R-grip = Right handgrip strength; DM drugs = Diabetes Meletus’s drugs; HT drugs = Hypertension’s drugs; OP = Osteoporosis. P≤0.05 means statistical significance.^
