# Supplementary material for: Diabetic Peripheral Neuropathy With Low Appendicular Muscle Mass Is Associated With Osteoporosis, Falls, and Fractures
Source: J Diabetes. 2026 Oct 28;18(11):e70276. doi: 10.1111/1753-0407.70276 (PMC13715079; doi:10.1111/1753-0407.70276)
Supplement: Supplementary file 2 [file JDB-18-e70276-s002.docx]

**Supplementary Table 4: Results from the COX Survival Analysis of the Local Database.**

| **Dependent Variables** | **Independent Variables** | **HR** | **95%CI_low** | **95%CI_high** | **P value** |
| --- | --- | --- | --- | --- | --- |
| OP | None | 1.000 | Ref. | Ref. | - |
|  | LAMM | 1.250 | 0.377 | 4.146 | 0.716 |
|  | DPN | 1.264 | 0.389 | 4.102 | 0.697 |
|  | LAMM+DPN | 0.651 | 0.055 | 7.688 | 0.733 |
| Falls | None | 1.000 | Ref. | Ref. | - |
|  | LAMM | 2.517 | 1.154 | 5.494 | 0.020 |
|  | DPN | 0.443 | 0.140 | 1.398 | 0.165 |
|  | LAMM+DPN | 2.409 | 0.596 | 9.732 | 0.217 |
| Fractures | None | 1.000 | Ref. | Ref. | - |
|  | LAMM | 0.798 | 0.211 | 3.021 | 0.740 |
|  | DPN | 0.265 | 0.030 | 2.332 | 0.231 |
|  | LAMM+DPN | / | / | / | / |

^Notes: Data at the baseline moment deleted the outcome events that had already occurred. "None" refers to the patient group without diabetic peripheral neuropathy and without low appendicular muscle mass.; "LAMM" refers to the patient group with only low appendicular muscle mass; “DPN” refers to the group of patients with only^ ^clinically diagnosed diabetic peripheral neuropathy; “LAMM+DPN” means the comorbid low appendicular muscle mass and^ ^clinically diagnosed diabetic peripheral neuropathy group. The reference group within the group is based on the level of "None". P≤0.05 is significantly significant. It is difficult for the LAMM+DPN group to make an accurate estimate. Adjusted for the following variables: age, course of DM, BMI, WHtR, BF, UACR, TC, TG, ALB, eGFR, HbA1c, VD, TP1NP, CTX, gender, DR, DKD, DF, metformin, other hypoglycemic drugs, thiazolidinedione, SGLT2i, DPP4i, GLP1ra, ACEI/ARB, statins, smoking, HT. HR, hazard ratio; CI, confidence interval; OP,^ ^Osteoporosis.^
