# Supplementary material for: Diabetic Peripheral Neuropathy With Low Appendicular Muscle Mass Is Associated With Osteoporosis, Falls, and Fractures
Source: J Diabetes. 2026 Oct 28;18(11):e70276. doi: 10.1111/1753-0407.70276 (PMC13715079; doi:10.1111/1753-0407.70276)
Supplement: Supplementary file 3 [file JDB-18-e70276-s003.docx]

**Supplementary Table 2:**

**Results from the Firth-penalized Binary Logistic Regression Analysis of the Local Database.**

| **Dependent Variables** | **Independent Variables** | **OR** | **95%CI_low** | **95%CI_high** | **P value** |
| --- | --- | --- | --- | --- | --- |
| OP | None | Ref. | Ref. | Ref. | Ref. |
|  | LAMM | 1.783 | 1.495 | 2.126 | ＜0.001 |
|  | DPN | 1.259 | 1.009 | 1.566 | 0.040 |
|  | LAMM+DPN | 2.151 | 1.645 | 2.805 | ＜0.001 |
| Falls | None | Ref. | Ref. | Ref. | Ref. |
|  | LAMM | 2.399 | 1.888 | 3.052 | ＜0.001 |
|  | DPN | 1.440 | 1.034 | 1.980 | 0.027 |
|  | LAMM+DPN | 4.667 | 3.406 | 6.376 | ＜0.001 |
| Fractures | None | Ref. | Ref. | Ref. | Ref. |
|  | LAMM | 1.840 | 1.417 | 2.387 | ＜0.001 |
|  | DPN | 1857 | 1.365 | 2.507 | ＜0.001 |
|  | LAMM+DPN | 2.416 | 1.367 | 3.515 | ＜0.001 |

^Notes: The reference group within the group is based on the level of "None". P≤0.05 is significantly significant. Adjusted for the following variables: age, course of DM, BMI, WHtR, BF, UACR, TC, TG, ALB, eGFR, HbA1c, VD, total PINP, CTX, gender, DR, DKD, DF, metformin, other hypoglycemic drugs, thiazolidinedione, SGLT2i, DPP4i, GLP1r, ACEI/ARB, statins, smoking, HT. "None" refers to the patient group without diabetic peripheral neuropathy and without low appendicular muscle mass.; "LAMM" refers to the patient group with only low appendicular muscle mass; “DPN” refers to the group of patients with only^ ^clinically diagnosed diabetic peripheral neuropathy; “LAMM+DPN” means the comorbid low appendicular muscle mass and^ ^clinically diagnosed diabetic peripheral neuropathy group. Course of DM = Course of diabetes mellitus; BMI = Body mass index; WHtR = Waist-to-height ratio; BF = Body fat ratio, UACR = Urinary albumin-to-creatinine ratio, TC = total cholesterol; TG = triglycerides; ALB =^ ^Albumin; eGFR = Estimated glomerular filtration rate; HbA1c = Glycated hemoglobin A1c; VD = 25-hydroxyvitamin D; total PINP = Type I procollagen N-terminal propeptide; CTX = Type I collagen cross-linked C-telopeptide; DR = Diabetic retinopathy; DKD = Diabetic kidney disease; DF = Diabetic foot; SGLT2i = Sodium-glucose linked transporter 2 inhibitors; DPP4i = Dipeptidyl peptidase-4 inhibitors; GLP1ra = Glucagon-like peptide-1 receptor agonists; ACEI/ARB = Angiotensin-converting enzyme inhibitors or Angiotensin II receptor blockers; HT = Hypertension; OP = Osteoporosis; OR = Odds ratio.^
