# Supplementary material for: Diabetic Peripheral Neuropathy With Low Appendicular Muscle Mass Is Associated With Osteoporosis, Falls, and Fractures
Source: J Diabetes. 2026 Oct 28;18(11):e70276. doi: 10.1111/1753-0407.70276 (PMC13715079; doi:10.1111/1753-0407.70276)
Supplement: Supplementary file 4 [file JDB-18-e70276-s006.docx]

**Supplementary Table 3:**

**Baseline and follow-up demographic characteristics of 651 patients.**

| **Variables** | | **Total（651）** | **None（353）** | **LAMM（187）** | **DPN（70）** | **LAMM+DPN（41）** | **P value** |
| --- | --- | --- | --- | --- | --- | --- | --- |
| Age (years) | Baseline | 61.00 (53.00-68.00) | 60.00 (51.00-67.00) | 61.00 (53.00-68.00) | 63.00 (56.00-68.00) | 68.00 (59.00-75.00) | 0.002 |
|  | Endpoint | 65.00 (57.00-72.00) | 64.00 (56.00-71.00) | 65.00 (58.00-72.00) | 65.50 (59.00-71.00) | 69.00 (63.00-76.00) | 0.008 |
| Male | Baseline | 341 (52.4%) | 174 (49.3%) | 113 (60.4%) | 25 (35.7%) | 29 (70.7%) | ＜0.001 |
|  | Endpoint | 341 (52.4%) | 174 (49.3%) | 113 (60.4%) | 25 (35.7%) | 29 (70.7%) | ＜0.001 |
| Course of DM (years) | Baseline | 8.00 (3.00-13.00) | 9.00 (3.00-13.00) | 7.00 (3.00-12.00) | 10.00 (5.25-13.75) | 7.00 (4.00-16.00) | 0.123 |
|  | Endpoint | 11.00 (7.50-18.00) | 11.00 (8.00-18.00) | 12.00 (6.00-17.00) | 13.50 (10.00-20.00) | 11.00 (8.00-20.00) | 0.342 |
| BMI (kg/m^2^) | Baseline | 24.86 (22.66-27.28) | 25.71 (23.73-27.70) | 22.96 (21.16-25.57) | 25.41 (23.02-27.92) | 23.53 (20.70-25.01) | ＜0.001 |
|  | Endpoint | 24.77 (22.62-27.03) | 25.51 (24.31-27.34) | 22.62 (20.93-24.77) | 26.20 (23.80-27.80) | 22.23 (21.34-24.78) | ＜0.001 |
| WHtR | Baseline | 0.54 (0.51-0.58) | 0.55 (0.52-0.59) | 0.52 (0.48-0.55) | 0.54 (0.51-0.61) | 0.52 (0.47-0.56) | ＜0.001 |
|  | Endpoint | 0.54 (0.52-0.58) | 0.56 (0.53-0.59) | 0.53 (0.49-0.55) | 0.58 (0.54-0.62) | 0.52 (0.50-0.56) | ＜0.001 |
| BF | Baseline | 0.28 ± 0.08 | 0.30 ± 0.08 | 0.25 ± 0.09 | 0.29 ± 0.08 | 0.23 ± 0.08 | ＜0.001 |
|  | Endpoint | 0.29 (0.24-0.35) | 0.30 (0.26-0.37) | 0.28 (0.21-0.32) | 0.32 (0.27-0.37) | 0.24 (0.20-0.31) | ＜0.001 |
| HT | Baseline | 395 (60.7%) | 216 (61.2%) | 111 (59.4%) | 43 (61.4%) | 25 (61.0%) | 0.979 |
|  | Endpoint | 466 (71.6%) | 254 (72.0%) | 122 (65.2%) | 56 (80.0%) | 34 (82.9%) | 0.033 |
| Smoking | Baseline | 129 (19.8%) | 66 (18.7%) | 46 (24.6%) | 8 (11.4%) | 9 (22.0%) | 0.103 |
|  | Endpoint | 154 (23.7%) | 76 (21.5%) | 55 (29.4%) | 10 (14.3%) | 13 (31.7%) | 0.027 |
| Metformin | Baseline | 318 (48.8%) | 160 (45.3%) | 87 (46.5%) | 45 (64.3%) | 26 (63.4%) | 0.006 |
|  | Endpoint | 371 (57.0%) | 170 (48.2%) | 122 (65.2%) | 46 (65.7%) | 33 (80.5%) | ＜0.001 |
| Thiazolidinedione | Baseline | 80 (12.3%) | 41 (11.6%) | 22 (11.8%) | 8 (11.4%) | 9 (22.0%) | 0.284 |
|  | Endpoint | 60 (9.2%) | 32 (9.1%) | 15 (8.0%) | 7 (10.0%) | 6 (14.6%) | 0.611 |
| SGLT2i | Baseline | 10 (1.5%) | 8 (2.3%) | 2 (1.1%) | 0 (0.0%) | 0 (0.0%) | 0.611 |
|  | Endpoint | 91 (14.0%) | 61 (17.3%) | 30 (16.0%) | 0 (0.0%) | 0 (0.0%) | ＜0.001 |
| DPP4i | Baseline | 52 (8.0%) | 29 (8.2%) | 20 (10.7%) | 1 (1.4%) | 2 (4.9%) | 0.068 |
|  | Endpoint | 208 (32.0%) | 121 (34.3%) | 77 (41.2%) | 5 (7.1%) | 5 (12.2%) | ＜0.001 |
| GLP1ra | Baseline | 21 (3.2%) | 14 (4.0%) | 7 (3.7%) | 0 (0.0%) | 0 (0.0%) | 0.251 |
|  | Endpoint | 180 (27.6%) | 112 (31.7%) | 68 (36.4%) | 0 (0.0%) | 0 (0.0%) | ＜0.001 |
| Other hypoglycemic drugs | Baseline | 458 (70.4%) | 247 (70.0%) | 126 (67.4%) | 51 (72.9%) | 34 (82.9%) | 0.247 |
|  | Endpoint | 501 (77.0%) | 267 (75.6%) | 143 (76.5%) | 54 (77.1%) | 37 (90.2%) | 0.195 |
| ACEI/ARB | Baseline | 144 (22.1%) | 77 (21.8%) | 40 (21.4%) | 13 (18.6%) | 14 (34.1%) | 0.258 |
|  | Endpoint | 254 (39.0%) | 128 (36.3%) | 71 (38.0%) | 34 (48.6%) | 21 (51.2%) | 0.091 |
| Statins | Baseline | 81 (12.4%) | 51 (14.4%) | 23 (12.3%) | 2 (2.9%) | 5 (12.2%) | 0.038 |
|  | Endpoint | 286 (43.9%) | 175 (49.6%) | 93 (49.7%) | 9 (12.9%) | 9 (22.0%) | ＜0.001 |
| HbA1c (%) | Baseline | 8.60 (7.10-10.70) | 8.30 (7.10-10.70) | 8.70 (7.05-10.60) | 9.25 (7.53-10.85) | 8.90 (6.90-10.60) | 0.620 |
|  | Endpoint | 8.10 (6.80-9.85) | 8.00 (6.90-9.80) | 8.10 (6.70-10.00) | 8.15 (7.12-9.67) | 8.00 (6.40-10.40) | 0.965 |
| ALB (g/L) | Baseline | 40.30 (37.60-42.90) | 40.95 (38.40-43.70) | 39.50 (36.80-42.40) | 38.65 (36.92-42.18) | 38.90 (36.60-40.50) | ＜0.001 |
|  | Endpoint | 40.30 (37.55-42.95) | 40.90 (38.40-43.30) | 40.00 (37.60-42.70) | 38.75 (35.83-41.50) | 37.40 (34.50-40.00) | ＜0.001 |
| TC (mmol/L) | Baseline | 4.55 (3.83-5.34) | 4.62 (3.85-5.36) | 4.53 (3.84-5.22) | 4.45 (3.89-5.39) | 4.20 (3.63-5.29) | 0.620 |
|  | Endpoint | 4.20 (3.51-4.97) | 4.29 (3.60-5.06) | 4.12 (3.50-4.80) | 4.11 (3.56-4.75) | 3.64 (3.20-5.17) | 0.233 |
| TG (mmol/L) | Baseline | 1.42 (0.95-2.12) | 1.60 (1.03-2.28) | 1.21 (0.85-1.75) | 1.35 (1.01-1.91) | 1.06 (0.89-1.59) | ＜0.001 |
|  | Endpoint | 1.34 (0.94-1.99) | 1.49 (1.09-2.10) | 1.22 (0.89-1.65) | 1.09 (0.85-1.78) | 1.07 (0.76-1.72) | ＜0.001 |
| eGFR (ml/min) | Baseline | 98.93 (88.13-109.21) | 99.30 (88.49-109.20) | 100.88 (91.42-110.99) | 93.78 (81.41-105.15) | 93.85 (76.90-106.94) | 0.001 |
|  | Endpoint | 94.68 (78.44-104.54) | 95.47 (78.65-104.92) | 95.15 (86.07-107.61) | 89.33 (64.94-100.45) | 90.92 (77.54-98.04) | 0.008 |
| Total PINP (ng/mL) | Baseline | 39.94 (31.27-40.68) | 39.94 (30.09-41.26) | 39.81 (30.81-40.49) | 40.14 (39.85-40.45) | 39.94 (31.44-40.43) | 0.132 |
|  | Endpoint | 37.73 (26.93-44.44) | 38.31 (27.99-44.26) | 36.61 (24.86-43.30) | 39.76 (29.51-50.57) | 34.48 (26.31-44.53) | 0.351 |
| CTX (ng/mL) | Baseline | 0.27 (0.20-0.42) | 0.28 (0.20-0.45) | 0.28 (0.22-0.42) | 0.25 (0.21-0.39) | 0.26 (0.18-0.33) | 0.301 |
|  | Endpoint | 0.32 (0.22-0.47) | 0.32 (0.22-0.45) | 0.33 (0.23-0.48) | 0.32 (0.25-0.47) | 0.39 (0.20-0.51) | 0.401 |
| VD (ng/mL) | Baseline | 22.27 (20.25-23.41) | 22.26 (19.47-23.76) | 22.30 (20.90-25.55) | 22.26 (20.70-22.36) | 22.25 (22.17-22.39) | 0.311 |
|  | Endpoint | 22.60 (18.63-29.32) | 22.68 (19.53-29.34) | 22.97 (20.27-32.04) | 22.24 (16.05-26.95) | 18.88 (14.79-25.48) | 0.001 |
| UACR (mg/g) | Baseline | 13.29 (6.45-61.98) | 12.21 (6.22-42.49) | 13.10 (5.73-47.71) | 18.40 (10.10-100.66) | 15.42 (7.26-145.17) | 0.008 |
|  | Endpoint | 15.80 (6.35-95.20) | 15.00 (6.20-92.12) | 12.79 (5.29-53.28) | 32.77 (9.20-183.59) | 32.65 (7.67-258.32) | 0.002 |
| DR | Baseline | 123 (18.9%) | 66 (18.7%) | 29 (15.5%) | 20 (28.6%) | 8 (19.5%) | 0.127 |
|  | Endpoint | 198 (30.4%) | 112 (31.7%) | 45 (24.1%) | 27 (38.6%) | 14 (34.1%) | 0.097 |
| DKD | Baseline | 222 (34.1%) | 117 (33.1%) | 57 (30.5%) | 30 (42.9%) | 18 (43.9%) | 0.146 |
|  | Endpoint | 272 (41.8%) | 141 (39.9%) | 70 (37.4%) | 39 (55.7%) | 22 (53.7%) | 0.019 |
| DF | Baseline | 16 (2.5%) | 5 (1.4%) | 3 (1.6%) | 4 (5.7%) | 4 (9.8%) | 0.005 |
|  | Endpoint | 14 (2.2%) | 4 (1.1%) | 2 (1.1%) | 4 (5.7%) | 4 (9.8%) | 0.002 |
| OP | Baseline | 148 (22.7%) | 65 (18.4%) | 55 (29.4%) | 21 (30.0%) | 7 (17.1%) | 0.010 |
|  | Endpoint | 119 (18.3%) | 44 (12.5%) | 44 (23.5%) | 19 (27.1%) | 12 (29.3%) | ＜0.001 |
| Falls | Baseline | 33 (5.1%) | 11 (3.1%) | 15 (8.0%) | 2 (2.9%) | 5 (12.2%) | 0.011 |
|  | Endpoint | 90 (13.8%) | 23 (6.5%) | 43 (23.0%) | 11 (15.7%) | 13 (31.7%) | ＜0.001 |
| Fractures | Baseline | 45 (6.9%) | 26 (7.4%) | 12 (6.4%) | 3 (4.3%) | 4 (9.8%) | 0.684 |
|  | Endpoint | 72 (11.1%) | 24 (6.8%) | 26 (13.9%) | 15 (21.4%) | 7 (17.1%) | 0.001 |

^Notes: Patients were not subjected to any selection or intervention at baseline. P≤0.05 is significantly significant. "None" refers to the patient group without diabetic peripheral neuropathy and without low appendicular muscle mass.; "LAMM" refers to the patient group with only low appendicular muscle mass; “DPN” refers to the group of patients with only^ ^clinically diagnosed diabetic peripheral neuropathy; “LAMM+DPN” means the comorbid low appendicular muscle mass and^ ^clinically diagnosed diabetic peripheral neuropathy group. Course of DM = Course of diabetes mellitus; BMI = Body mass index; WHtR = Waist-to-height ratio; BF = Body fat ratio, UACR = Urinary albumin-to-creatinine ratio, TC = total cholesterol; TG = triglycerides; ALB = Albumin; eGFR = Estimated glomerular filtration rate; HbA1c = Glycated hemoglobin A1c; VD = 25-hydroxyvitamin D; Total PINP = Type I procollagen N-terminal propeptide; CTX = Type I collagen cross-linked C-telopeptide; DR = Diabetic retinopathy; DKD = Diabetic kidney disease; DF = Diabetic foot; SGLT2i = Sodium-glucose linked transporter 2 inhibitors; DPP4i = Dipeptidyl peptidase-4 inhibitors; GLP1ra = Glucagon-like peptide-1 receptor agonists; ACEI/ARB = Angiotensin-converting enzyme inhibitors or Angiotensin II receptor blockers; HT = Hypertension; OP = Osteoporosis. Continuous variables were expressed as “Mean ± SD” if normally distributed, or as “Median (IQR)” if non-normally distributed; categorical variables were presented as “n (%)”.^
