# Supplementary material for: Diabetic Peripheral Neuropathy With Low Appendicular Muscle Mass Is Associated With Osteoporosis, Falls, and Fractures
Source: J Diabetes. 2026 Oct 28;18(11):e70276. doi: 10.1111/1753-0407.70276 (PMC13715079; doi:10.1111/1753-0407.70276)
Supplement: Supplementary file 5 [file JDB-18-e70276-s007.docx]

**Supplementary Tables: Significant Serum Antibody-Reactive Peptides and Representative Annotations**

**Supplementary Table 5a. Differentially reactive peptides identified in the LAMM+DPN versus None comparison.**

| No. | Peptide ID | Gene/Protein annotation | Peptide sequence | log2FC | Fold change | P value | BH-FDR q value |
| --- | --- | --- | --- | --- | --- | --- | --- |
| 1 | #632706 | FOXP2 | QQQQQQQQQQQQQQQQQQQQQQQHPGKQAKEQQQQQQQQQLAAQQLVFQQQLLQMQ | 1.836 | 3.569 | 4.45e-06 | 0.0038 |
| 2 | #248649 | INAVA | SSRLPTQPGPGWSRCPPSLLCALSFQKSTMESKDEVSDTDSGIILQSGPDSPVSPM | 1.500 | 2.828 | 1.10e-05 | 0.0047 |
| 3 | #634931 | NP_001354759.1 | RPASLYLARSKSISSSNPDLAVAPGSVDDEVSRILASKGIDRSHSWVNSAYAPGGS | 1.355 | 2.558 | 3.32e-05 | 0.0094 |
| 4 | #422566 | UFD1 | TKPDKAVSIIECDMNVDFDAPLGYKEPERQVQHEESTEGEADHSGYAGELGFRAFS | 1.435 | 2.705 | 8.08e-05 | 0.0173 |
| 5 | #361115 | SLC43A2 | QPDAAVAPSFMHSVFSPILLLSLVTMCVTQLRLIFYMGAMNNILKFLVSGDQKTVG | 1.102 | 2.147 | 1.22e-04 | 0.0180 |
| 6 | #455062 | CIC | GDSSGEDPEGNKGFGRKVFSPVIRSSFTHCRPPLDPEPPGPPDPPVAFGKGYGSAP | 1.330 | 2.515 | 1.40e-04 | 0.0180 |
| 7 | #455063 | CIC | RKVFSPVIRSSFTHCRPPLDPEPPGPPDPPVAFGKGYGSAPSSSASSPASSSASAA | 1.528 | 2.884 | 1.51e-04 | 0.0180 |
| 8 | #167911 | PRDM15 | QPLVIVEDKEPTEQVAEIITEVPPDEPVSATPDERIMELVLGKLATTTTDTSSVPK | 1.232 | 2.349 | 1.69e-04 | 0.0180 |
| 9 | #547489 | ZMAT5 | CRKFLLTGQCDFGSNCRFSHMSERDLQELSIQVEEERRAREWLLDAPELPEGHLED | 1.242 | 2.366 | 4.32e-04 | 0.0410 |

**Note:** Differentially reactive peptides were defined as BH-FDR q < 0.05 and |log2FC| > 1 (equivalent to a fold change > 2 or < 0.5). Positive log2FC values indicate higher antibody reactivity in the LAMM+DPN group than in the None group. All nine significant peptides showed increased reactivity.

**Supplementary Table 5b. Representative gene/protein annotations corresponding to the significant peptides in the LAMM+DPN versus None comparison.**

| **No.** | **Gene/Protein annotation** | **Representative peptide ID** | **No. of significant peptides** | **Representative log2FC** | **Fold change** | **P value** | **BH-FDR q value** |
| --- | --- | --- | --- | --- | --- | --- | --- |
| 1 | FOXP2 | #632706 | 1 | 1.836 | 3.569 | 4.45e-06 | 0.0038 |
| 2 | INAVA | #248649 | 1 | 1.500 | 2.828 | 1.10e-05 | 0.0047 |
| 3 | NP_001354759.1 | #634931 | 1 | 1.355 | 2.558 | 3.32e-05 | 0.0094 |
| 4 | UFD1 | #422566 | 1 | 1.435 | 2.705 | 8.08e-05 | 0.0173 |
| 5 | SLC43A2 | #361115 | 1 | 1.102 | 2.147 | 1.22e-04 | 0.0180 |
| 6 | CIC | #455063 | 2 | 1.528 | 2.884 | 1.51e-04 | 0.0180 |
| 7 | PRDM15 | #167911 | 1 | 1.232 | 2.349 | 1.69e-04 | 0.0180 |
| 8 | ZMAT5 | #547489 | 1 | 1.242 | 2.366 | 4.32e-04 | 0.0410 |

**Note:** The representative peptide for each gene/protein annotation was selected from the peptide-level results. Nine significant peptides corresponded to eight representative annotations because CIC was represented by two significant peptides. “No. of significant peptides” was recalculated from the peptide-level significant results and does not refer to the total number of library peptides annotated to that gene/protein.
